# Structural Online Damage Identification and Dynamic Reliability Prediction Method Based on Unscented Kalman Filter

**DOI:** 10.3390/s24237582

**Published:** 2024-11-27

**Authors:** Yan Zhang, Yongbo Zhang, Jinhui Yu, Fei Zhao, Shihao Zhu

**Affiliations:** 1 School of Aeronautic Science and Engineering, Beihang University, Beijing 100191, China; 2 Aircraft and Propulsion Laboratory, Ningbo Institute of Technology, Beihang University, Ningbo 315100, China

**Keywords:** Unscented Kalman Filter, structural damage identification, dynamic reliability prediction, performance degradation process

## Abstract

As sensor monitoring technology continues to evolve, structural online monitoring and health management have found numerous applications across various fields. However, challenges remain concerning the real-time diagnosis of structural damage and the accuracy of dynamic reliability predictions. In this paper, a structural online damage identification and dynamic reliability prediction method based on Unscented Kalman Filter (UKF) is presented. Specifically, in the Wiener degradation process with random effects on structural performance, the structural damage identification is initially realized using UKF. Following that, the EM algorithm is employed for estimating the performance model parameters. Eventually, dynamic reliability prediction is realized based on conditional probability. The simulation results indicate that the method effectively estimates the damage state during the structure’s use while providing accurate, real-time, and dynamic reliability predictions for the system.

## 1. Introduction

Online structural monitoring and health management utilize modern information technology to diagnose and detect the damage state of a structure promptly, and to make real-time predictions and assessments of its reliability and safety [1]. “Online” means real-time or near real-time monitoring and evaluation of the health status of the structure, that is, the system can continuously collect data, analyze the data in a timely manner, and provide decision support and emergency response. The development of this technology has taken nearly fifty years; however, several key issues still limit its application, including the reliability and stability of the sensors, the challenges of real-time data acquisition and transmission, and the accuracy of the algorithm software.

Currently, the primary methods for dynamic damage identification using sensors include model modification [2,3], dynamic fingerprinting [4,5], genetic algorithms (GAs) [6,7], neural networks [8,9], wavelet analysis [10,11,12], and real-time signal processing methods [13,14,15,16]. The model modification pinpoints damage within the structure by comparing experimental vibration responses with theoretical calculations, utilizing optimized constraints to assess changes in stiffness. The dynamic fingerprinting method utilizes a relevant database to identify the corresponding damage state based on measured kinetic changes. The damage identification method based on GA uses the algorithm to find the relevant kinetic parameters that closely match the measured data to determine the location and extent of damage. Zhang [6] applied an improved generalized genetic algorithm to damage recognition; Ramezani Meysam [7] proposed an improved GA based on finite vibration modes to determine the damage severity and location in units and connections. Damage detection using neural networks establishes the relationship between key structural parameters and damage states to facilitate structural damage identification. Veronika [8] proposed a structural damage identification method of composite rotors based on fully connected neural networks and convolutional neural networks. Zhan [9] proposed a damage identification method in a beam-like structure using a strain FRF-based damage index and an artificial neural network. Wavelet analysis breaks down structural dynamic signals into valuable information for analyzing the state of structural damage.

Both the model modification and the dynamic fingerprinting method require testing and collecting substantial information, which limits their applicability in complex structures. GA and neural networks require substantial computational resources, exhibit slow convergence rates, and are prone to getting trapped in local minima. Wavelet analysis offers significant advantages for complex signals, but selecting the appropriate basis function can be challenging. The real-time signal processing method performs structural parameter identification by directly constructing equations from dynamic data in the time domain. This method helps prevent information loss that may occur during intermediate conversion processes, including the least squares estimation [13], the maximum likelihood estimation [14], and the Kalman filter method [15,16]. Kalman filter is a recursive linear minimum variance estimation method that uses the system’s state equations and observed data to optimally estimate the state, enabling physical parameter identification. It can integrate dynamic information and holds significant potential for multi-sensor data fusion.

Currently, various filter methods are actively used in the field of structural damage and reliability analysis, including the extended Kalman filter (EKF) [17,18], Unscented Kalman Filter (UKF) [19], particle filter (PF) [20], H∞ filter [21], Moving Horizon Estimation (MHE) [22], and Neural Network-Based Filters [23,24]. The EKF is a nonlinear extension of the Kalman filter that uses a Taylor series expansion to linearize the system’s nonlinear dynamics and observation models. However, this method is primarily applicable to linear or mildly nonlinear structures, and the calculation of the Jacobian matrix can be relatively complex. The UKF addresses nonlinear problems using the “unscented transformation”. Unlike the EKF, the UKF does not require the calculation of the Jacobian matrix, offers better adaptability to nonlinear systems, and can achieve higher estimation accuracy. The PF estimates a system’s state using a set of randomly sampled particles and is capable of handling highly nonlinear systems with non-Gaussian noise in parameter estimation. Cristiani [20] diagnosed and predicted the damage of composite double cantilever beam specimens through particle filtering and surrogate models. The H∞ filter designs the filter based on the criterion of minimizing error energy, making it well suited for handling situations with uncertainty and strong noise. Tang [21] used neural networks and H∞ filters to conduct structural damage detection. MHE is an optimization-based method that estimates the current system state by minimizing an error function within a sliding window. Neural Network-Based Filters, on the other hand, use deep learning models to estimate system states and excel in handling complex, nonlinear, or high-dimensional systems. Pathirage [23] proposed a structural damage identification framework based on autoencoders, which can support deep neural networks. Rautela [24] applied model-assisted convolutional and recurrent neural networks for structural damage detection and localization using ultrasonic-guided waves. However, methods like PF, H∞ filters, MHE, and Neural Network-Based Filters require substantial computing resources and memory, which limits their real-time performance. In contrast, the UKF offers significant advantages in both ensuring calculation accuracy and maintaining real-time performance for online damage identification and reliability prediction.

On the other hand, methods based on structural performance degradation analysis are currently the dominant approach for studying the dynamic reliability of systems. These methods primarily include regression analysis [25,26,27], time series analysis, stochastic process analysis [28,29,30], and other analytical techniques [31,32]. Regression analysis uses regression modeling to study the process of structural performance degradation. Time series analysis provides reliability predictions and assessments based directly on degraded data. Stochastic process analysis uses specific stochastic models to represent the problem. Common models include the inverse Gaussian process, Gamma process, and Wiener process. Pan [28] used the Wiener process to model the degradation of product performance parameters; Niu [30] accomplished the performance reliability degradation analysis and lifetime prediction based on the generalized normal gray cloud Bayesian Wiener process. There are also some other methods such as dynamic reliability assessment using fault trees and dynamic Bayesian networks [31] and probability density evolution methods [32].

The current research on system dynamic reliability prediction typically assumes that performance parameters are directly observable. However, in practice, the performance degradation process is generally implicit within the system, making direct monitoring difficult. At the same time, performance degradation is accompanied by uncertainty, as the system is exposed to varying environments, stresses, and other factors during operation. Therefore, to realize the real-time reliability prediction of the implied performance degradation process in complex systems, this paper proposes a structural online damage identification and dynamic reliability prediction method based on UKF. It is important to emphasize that, while UKF has been widely applied to nonlinear systems, this paper’s contribution lies in its tailored adaptation for structural damage identification. We integrate online damage detection with dynamic reliability assessment, specifically designed for systems with complex degradation processes. This approach enables the rapid identification of damage coefficients and provides accurate, real-time, and dynamic reliability assessments.

The rest of this article is organized as follows. Section 2 provides a specific description of the research problem and establishes the system equations for the implied performance degradation. Section 3 proposes an online damage identification and dynamic reliability prediction method for structures based on UKF. Section 4 carries out simulation experiments and discussion of the method with an example. Section 5 summarizes this article.

## 2. Problem Statement

### 2.1. Identification of Implicit Performance Degeneration Processes

For general nonlinear systems, there are

(1) $$\begin{array}{l} X_{k}=f(X_{k-1},\Phi ,t_{k-1})+w_{k-1}\\ Y_{k}=h(X_{k},t_{k})+v_{k} \end{array}$$

where $X_{k}$ is the state variable, $Y_{k}$ is the observation variable, and Φ is the parameter vector. Additionally, $w_{k}$ and $v_{k}$ correspond to the system noise and measurement noise, respectively. Assuming that the characteristic quantity reflecting the performance degradation is represented by φ, its relationship with the parameter vector can be expressed by

(2) Φ=u(φ).

In structural systems, state variables such as displacement, velocity, and acceleration can be directly observed. However, the stiffness coefficients of the structure, which serve as implied performance variables, are challenging to measure in real-time. This paper utilizes a Wiener process with random effects on the drift coefficients to model the performance degradation process.

Assume that the performance eigenvalue φ satisfies

(3) $$\varphi (\tau )=\eta_{0}+\eta \tau +\sigma B(\tau ),\eta \sim N(\mu_{\eta },{\sigma_{\eta }}^{2})$$

where $\eta_{0}=\varphi (0)$ is known; τ represents time; $\mu_{\eta }$, $\sigma_{\eta }$, and σ are unknown parameters; B(τ) is standard Brownian motion.

Brownian motion with drift satisfies $\varphi (\tau +\Delta \tau )-\varphi (\tau )\sim N(\eta \Delta \tau ,\sigma^{2}\Delta \tau )$. Discretization has

(4) $$\begin{array}{l} \varphi_{k}=\varphi_{k-1}+\eta T+\varepsilon_{k-1}\\ \varphi_{0}=\eta_{0} \end{array}$$

where T is the sampling step, $\varepsilon_{k}\sim N(0,\sigma^{2}T)$. Then, the system equations for the final implied performance degradation are

(5) $$\begin{array}{l} X_{k}=f(X_{k-1},u(\varphi ))+w_{k-1}\\ \varphi_{k}=\varphi_{k-1}+\eta T+\varepsilon_{k-1},\eta \sim N(\mu_{\eta },{\sigma_{\eta }}^{2})\\ Y_{k}=h(X_{k})+v. \end{array}$$

### 2.2. Dynamical System Equations for Structure Stiffness Degradation

The dynamical equation of the structure is

(6) $$M\ddot{x}(t)+C\dot{x}(t)+Kx(t)=Uf(t)$$

where M, C, and K represent the overall mass, damping, and stiffness matrices of the structure, respectively. U denotes the overall equivalent nodal load action vector. The vectors $\ddot{x}$, $\dot{x}$, and x represent acceleration, velocity, and displacement at a given time, while f(t) represents the time-dependent external excitation force.

Transforming Equation (6) into the form of a matrix, we have

(7) $$\left[\begin{array}{c} \dot{x}\\ \ddot{x} \end{array}\right]=\left[\begin{array}{cc} 0 & I\\ -M\backslash K & -M\backslash C \end{array}\right]\left[\begin{array}{c} x\\ \dot{x} \end{array}\right]+\left[\begin{array}{c} 0\\ M\backslash U \end{array}\right]f(t).$$

Given $X={\left[\begin{array}{cc} x & \dot{x} \end{array}\right]}^{T}$, $A=\left[\begin{array}{cc} 0 & I\\ -M\backslash K & -M\backslash C \end{array}\right]$, $B=\left[\begin{array}{c} 0\\ M\backslash U \end{array}\right]$, it can be obtained

(8) $$\dot{X}=AX+Bf(t).$$

Discretization has

(9) $$X_{k}=F_{k-1}X_{k-1}+U_{k-1}f_{k-1}$$

where

(10) $$F_{k-1}=e^{A(\Delta t)}$$

(11) $$U_{k-1}=\int_{t_{k-1}}^{t_{k}}e^{A(t_{k}-\tau )}d\tau B=[I-e^{-A\left(\Delta t\right)}]A^{-1}B.$$

$X_{k}$ of Equation (9) contains the displacement vector and the velocity vector. If the stiffness of the structure is selected as the characteristic parameter for damage performance, a damage coefficient is applied to the stiffness matrix of each element to quantify the extent of damage. Let the element stiffness matrix be denoted as $K^{e}$, with the corresponding stiffness damage coefficient represented by xk. The variation of the stiffness matrix with time t is

(12) $$K^{e}(t)=K^{e}xk(t).$$

Setting the variable $XK=[xk_{1},\cdots ,xk_{n}]$ and increasing its dimension to include it in the state vector, we have

(13) $$\left[\begin{array}{c} X_{k}\\ XK_{k} \end{array}\right]=\left[\begin{array}{cc} F_{k-1} & 0\\ 0 & I \end{array}\right]\left[\begin{array}{c} X_{k-1}\\ XK_{k-1} \end{array}\right]+\left[\begin{array}{c} U_{k-1}\\ 0 \end{array}\right]f_{k-1}.$$

In dynamics, measurement equations primarily utilize displacement, velocity, or acceleration as measurement signals. When acceleration sensors are employed, the following measurement equation is

(14) $$Z_{k}={\ddot{x}}_{k}=M^{-1}\left[Uf(t)-C\dot{x}(t)-Kx(t)\right]$$

In light of the performance degradation process discussed in Section 2.1, the structural system equations analyzed in this paper are

(15) $$\left[\begin{array}{c} X_{k}\\ XK_{k} \end{array}\right]=\left[\begin{array}{cc} F_{k-1} & 0\\ 0 & I \end{array}\right]\left[\begin{array}{c} X_{k-1}\\ XK_{k-1} \end{array}\right]+\left[\begin{array}{c} U_{k-1}\\ 0 \end{array}\right]f_{k-1}+w_{k}$$

(16) $$xk_{k}=xk_{k-1}+\eta \Delta t+\varepsilon_{k-1}$$

(17) $$Z_{k}=M^{-1}\left[Uf(t)-C\dot{x}(t)-Kx(t)\right]+v_{k}$$

where $\left\{w_{k}\right\}$ and $\left\{v_{k}\right\}$ are system noise and measurement noise, $\eta \sim N(\mu_{\eta },{\sigma_{\eta }}^{2})$,$\varepsilon \sim N(0,\sigma^{2})$.

## 3. Methodology

### 3.1. Damage Parameter Estimation by UKF

The equations for identifying the structural damage parameters are

(18) $$\left[\begin{array}{c} X_{k}\\ XK_{k} \end{array}\right]=\left[\begin{array}{cc} F_{k-1} & 0\\ 0 & I \end{array}\right]\left[\begin{array}{c} X_{k-1}\\ XK_{k-1} \end{array}\right]+\left[\begin{array}{c} U_{k-1}\\ 0 \end{array}\right]f_{k-1}+w_{k}$$

(19) $$Y_{k}=[{\begin{array}{cc} -M\backslash K & -M\backslash C]X_{k}+M\backslash U_{k}f \end{array}}_{k}+v_{k}$$

(20) $$\begin{array}{l} E(w_{k})=0,E(w_{i}w_{j}^{T})=Q_{k}\delta_{ij};\\ E(v_{k})=0,E(v_{i}v_{j}^{T})=R_{k}\delta_{ij};\\ E(w_{i}v_{j}^{T})=0 \end{array}$$

where (18) is the state equation and (19) is the measurement equation; $\left\{w_{k}\right\}$ and $\left\{v_{k}\right\}$ are zero-mean, uncorrelated white noise processes with known covariance matrices $Q_{k}$ and $R_{k}$, respectively; $\delta_{ij}$ is the Kronecker-δ function.

The process of damage parameter estimation using UKF [33] is as follows:

Set $X_{a}={\left[X,XK\right]}^{T}$. The filter is initialized with the predicted mean and covariance of the state.

(21) $${\hat{X}}_{a,0}^{+}=E(X_{a,0})$$

(22) $$P_{0}^{+}=E\left[(X_{a,0}-{\hat{X}}_{a,0}^{+}){\left(X_{a,0}-{\hat{X}}_{a,0}^{+}\right)}^{T}\right]$$

where $X_{a,0}$, ${\hat{X}}_{a,0}^{+}$, and $P_{0}^{+}$ denote the initial state vector, the estimate of the initial state vector, and the covariance of the initial state vector, respectively.

**The equations for updating time.**

To propagate from step (k−1) to step k, the 2n sampling sigma points are calculated by the unscented transformation rule, as follows:

(23) $${\hat{X}}_{a,k-1}^{\left(i\right)}={\hat{X}}_{a,k-1}^{+}+{{\tilde{X}}_{a}}^{\left(i\right)},i=1,\cdots ,2n$$

(24) $${{\tilde{X}}_{a}}^{\left(i\right)}={\left(\sqrt{nP_{k-1}^{+}}\right)}_{i}^{T},i=1,\cdots ,n$$

(25) $${{\tilde{X}}_{a}}^{\left(n+i\right)}=-{\left(\sqrt{nP_{k-1}^{+}}\right)}_{i}^{T},i=1,\cdots ,n$$

where ${\hat{X}}_{a,k-1}^{+}$ and $P_{k-1}^{+}$ denote the posteriori mean and covariance of step (k−1), and ${\hat{X}}_{a,k-1}^{\left(i\right)}$ denotes the i-th sigma point.

Convert the sigma points to ${\hat{X}}_{a,k}^{\left(i\right)}$ by Equation (18), as follows:

(26) $${\hat{X}}_{a,k}^{\left(i\right)}=\left[\begin{array}{c} {\hat{X}}^{\left(i\right)}(k)\\ {\hat{XK}}^{\left(i\right)}(k) \end{array}\right]=\left[\begin{array}{cc} {\hat{F}}^{\left(i\right)}(k-1) & 0\\ 0 & I \end{array}\right]\left[\begin{array}{c} {\hat{X}}^{\left(i\right)}(k-1)\\ {\hat{XK}}^{\left(i\right)}(k-1) \end{array}\right]+\left[\begin{array}{c} {\hat{U}}^{\left(i\right)}(k-1)\\ 0 \end{array}\right]f(k-1).$$

The prior state estimate ${\hat{X}}_{a,k}^{-}$ at step k is obtained by weighted averaging:

(27) $${\hat{X}}_{a,k}^{-}=\frac{1}{2n}\sum_{i=1}^{2n}{\hat{X}}_{a,k}^{\left(i\right)}.$$

Calculate the prior covariance matrix $P_{k}^{-}$ of the state prediction by

(28) $$P_{k}^{-}=\frac{1}{2n}\sum_{i=1}^{2n}({\hat{X}}_{a,k}^{\left(i\right)}-{\hat{X}}_{a,k}^{-}){\left({\hat{X}}_{a,k}^{\left(i\right)}-{\hat{X}}_{a,k}^{-}\right)}^{T}+Q_{k-1}.$$

**The equations for updating measurement.**

Generate new sigma points using ${\hat{X}}_{a,k}^{-}$ and $P_{k}^{-}$ by

(29) $${\hat{X}}_{a,k}^{\left(i\right)}={\hat{X}}_{a,k}^{-}+{{\tilde{X}}_{a}}^{\left(i\right)},i=1,\cdots ,2n$$

(30) $${{\tilde{X}}_{a}}^{\left(i\right)}={\left(\sqrt{nP_{k}^{-}}\right)}_{i}^{T},i=1,\cdots ,n$$

(31) $${{\tilde{X}}_{a}}^{\left(n+i\right)}=-{\left(\sqrt{nP_{k}^{-}}\right)}_{i}^{T},i=1,\cdots ,n$$

where ${\hat{X}}_{a,k}^{\left(i\right)}$ denotes the i-th sigma point at step k.

Convert the sigma points to ${\hat{Y}}_{k}^{\left(i\right)}$ by Equation (19), as follows:

(32) $${\hat{Y}}_{k}^{\left(i\right)}=[{\begin{array}{cc} -M\backslash K_{k} & -M\backslash C_{k}]{\hat{X}}_{a,k}^{\left(i\right)}+M\backslash U_{k}f \end{array}}_{k},i=1,\cdots ,2n.$$

The measurement prediction ${\hat{Y}}_{k}$ and the covariance $P_{y}$ are calculated based on the statistics of the transformed sigma points, as follows:

(33) $${\hat{Y}}_{k}=\frac{1}{2n}\sum_{i=0}^{2n}{\hat{Y}}_{k}^{\left(i\right)}$$

(34) $$P_{y}=\frac{1}{2n}\sum_{i=0}^{2n}({\hat{Y}}_{k}^{\left(i\right)}-{\hat{Y}}_{k}){\left({\hat{Y}}_{k}^{\left(i\right)}-{\hat{Y}}_{k}\right)}^{T}+R_{k}.$$

The cross-correlation covariance, ${\hat{P}}_{xy}$, is calculated using

(35) $${\hat{P}}_{xy}=\frac{1}{2n}\sum_{i=0}^{2n}({\hat{X}}_{a,k}^{\left(i\right)}-{\hat{X}}_{a,k}^{-}){\left({\overset{⌢}{Y}}_{k}^{\left(i\right)}-{\hat{Y}}_{k}\right)}^{T}.$$

The Kalman gain matrix $D_{k}$ is approximated from the cross-correlation and measurement covariances, as follows:

(36) $$D_{k}=P_{xy}P_{y}^{-1}.$$

Calculate the posteriori estimated mean ${\hat{X}}_{a,k}^{+}$ and covariance $P_{k}^{+}$ of step k, completing the measurement update, as follows:

(37) $${\hat{X}}_{a,k}^{+}={\hat{X}}_{a,k}^{-}+D_{k}(Y_{k}-{\hat{Y}}_{k})$$

(38) $$P_{k}^{+}=P_{k}^{-}-D_{k}P_{y}D_{k}^{T}.$$

Through the above process, the state estimates at each step can be derived, enabling the parameter identification.

### 3.2. EM Algorithm

Based on the Wiener degradation process with random effects, the damage coefficient xk satisfies

(39) $$xk(t)=\eta_{0}+\eta t+\sigma B(t),\eta \sim N(\mu_{\eta },{\sigma_{\eta }}^{2}).$$

where η is the drift coefficient representing the degradation of structural stiffness damage performance, which follows a normal distribution with mean $\mu_{\eta }$ and variance ${\sigma_{\eta }}^{2}$; σ is the diffusion coefficient; and B(t) denotes the standard Brownian motion.

Discretization has

(40) $$\begin{array}{l} xk_{k}=xk_{k-1}+\eta \Delta t+\varepsilon_{k-1}\\ \eta \sim N(\mu_{\eta },{\sigma_{\eta }}^{2}),\varepsilon_{k-1}\sim N(0,\sigma^{2}). \end{array}$$

The parameters to be estimated in this degradation model include $\mu_{\eta }$, ${\sigma_{\eta }}^{2}$, and $\sigma^{2}$. The parameters include hidden variables, which cannot be directly estimated using the maximum likelihood method. The Expectation–Maximization (EM) algorithm is commonly used to solve the parameter estimation problem involving hidden variables in probabilistic models. Let $\Theta ={\left(\mu_{\eta },{\sigma_{\eta }}^{2},\sigma^{2}\right)}^{T}$ denote the parameter vector; the EM algorithm [34] is required, as the model contains hidden variables.

Set Z to be the overall degradation dataset, with a total of n data sets, each with m time points. Let $\Theta^{k}={\left({\mu_{\eta }}^{k},{\left({\sigma_{\eta }}^{2}\right)}^{k},{\left(\sigma^{2}\right)}^{k}\right)}^{T}$ be the estimate of the parameters at step k. Given $\Theta^{k}$, there is

(41) $$P(Z_{i}|\eta_{i},\Theta^{k})=\prod_{j=1}^{Z_{i}}f(\Delta xk_{ij}|\Theta^{k}).$$

Given that $\eta_{i}\sim N(\mu_{\eta },{\sigma_{\eta }}^{2})$, the posterior estimate of $\eta_{i}$ follows normal distribution, provided that $Z_{i}$ and $\Theta^{k}$ are known. Let its mean be ${\mu_{i}}^{k}$ and its variance be ${\left({\sigma_{i}}^{2}\right)}^{k}$. According to Bayes’ theorem, we can derive

(42) $$\begin{array}{ll} P(\eta_{i}|Z_{i},\Theta^{k}) & \propto P(Z_{i}|\eta_{i},\Theta^{k})P(\eta_{i}|\Theta^{k})\\ & \propto \exp\left[-\sum_{j=1}^{m}\frac{{\left(\Delta xk_{ij}-\eta_{i}\Delta t_{j}\right)}^{2}}{2{\left(\sigma^{2}\right)}^{k}\Delta t_{j}}\right]\exp\left[-\frac{{\left(\eta_{i}-\mu_{\eta }^{k}\right)}^{2}}{2{\left({\sigma_{\eta }}^{2}\right)}^{k}}\right]\\ & \propto \exp\left\{-\frac{{\left[\eta_{i}-\left((\sum_{j=1}^{m}\Delta xk_{ij}){\left({\sigma_{\eta }}^{2}\right)}^{k}+\mu_{\eta }^{k}{\left(\sigma^{2}\right)}^{k}\right)/\left((\sum_{j=1}^{m}\Delta t_{j}){\left({\sigma_{\eta }}^{2}\right)}^{k}+{\left(\sigma^{2}\right)}^{k}\right)\right]}^{2}}{2{\left(\sigma^{2}\right)}^{k}{\left({\sigma_{\eta }}^{2}\right)}^{k}/\left((\sum_{j=1}^{m}\Delta t_{j}){\left({\sigma_{\eta }}^{2}\right)}^{k}+{\left(\sigma^{2}\right)}^{k}\right)}\right\}. \end{array}$$

The posterior estimate of $\eta_{i}$ follows a normal distribution and can be obtained as

(43) $$P(\eta_{i}|Z_{i},\Theta^{k})=\frac{1}{\sqrt{2\pi {\left({\sigma_{i}}^{2}\right)}^{k}}}\exp\left[-\frac{{\left(\eta_{i}-\mu_{i}^{k}\right)}^{2}}{2{\left({\sigma_{i}}^{2}\right)}^{k}}\right]$$

where

(44) $${\mu_{i}}^{k}=\frac{(\sum_{j=1}^{m}\Delta xk_{ij}){\left({\sigma_{\eta }}^{2}\right)}^{k}+{\mu_{\eta }}^{k}{\left(\sigma^{2}\right)}^{k}}{(\sum_{j=1}^{m}\Delta t_{j}){\left({\sigma_{\eta }}^{2}\right)}^{k}+{\left(\sigma^{2}\right)}^{k}}$$

(45) $${\left(\sigma_{i}^{2}\right)}^{k}=\frac{2{\left(\sigma^{2}\right)}^{k}{\left({\sigma_{\eta }}^{2}\right)}^{k}}{(\sum_{j=1}^{m}\Delta t_{j}){\left({\sigma_{\eta }}^{2}\right)}^{k}+{\left(\sigma^{2}\right)}^{k}}.$$

Under the conditions of knowing $Z_{i}$ and $\Theta^{k}$, the EM algorithm first obtains

(46) $$\begin{array}{ll} Q(\Theta |Y,\Theta^{k})= & -\frac{1}{2}\sum_{i=1}^{n}[(m+1)\ln2\pi +\sum_{j=1}^{m}\ln\Delta t_{j}+m\ln\sigma^{2}+\ln{\sigma_{\eta }}^{2}\\ & +\sum_{j=1}^{m}\frac{{\left(\Delta xk_{ij}\right)}^{2}-2\mu_{i}^{k}\Delta xk_{ij}\Delta t_{j}+{\left(\Delta t_{j}\right)}^{2}({\left(\mu_{i}^{k}\right)}^{2}+{\left({\sigma_{i}}^{2}\right)}^{k})}{\sigma^{2}\Delta t_{j}}\\ & +\frac{{\left(\mu_{i}^{k}\right)}^{2}+{\left({\sigma_{i}}^{2}\right)}^{k}-2\mu_{i}^{k}\mu_{\eta }+{\mu_{\eta }}^{2}}{{\sigma_{\eta }}^{2}}]. \end{array}$$

At the M-step, setting $\frac{\partial Q}{\partial \Theta }=0$, the latest estimated value of parameter Θ is determined, and the final solution is

(47) $$\mu_{\eta }^{k+1}=\frac{1}{n}\sum_{i=1}^{n}\mu_{i}^{k}$$

(48) $${\left(\sigma_{\eta }^{2}\right)}^{k+1}=\frac{1}{n}\sum_{i=1}^{n}\left[{\left(\mu_{i}^{k}\right)}^{2}+{\left(\sigma_{i}^{2}\right)}^{k}-2\mu_{i}^{k}\mu_{\eta }^{k+1}+{\left(\mu_{\eta }^{k+1}\right)}^{2}\right]$$

(49) $${\left(\sigma^{2}\right)}^{k+1}=\frac{1}{nm}\sum_{i=1}^{n}\sum_{j=1}^{m}\frac{{\left(\Delta xk_{ij}\right)}^{2}-2\mu_{i}^{k}\Delta xk_{ij}\Delta t_{j}+{\left(\Delta t_{j}\right)}^{2}({\left(\mu_{i}^{k}\right)}^{2}+{\left(\sigma_{i}^{2}\right)}^{k})}{\Delta t_{j}}.$$

The EM algorithm operates through two fundamental steps, the E-step and the M-step, and iterates multiple times until a predefined convergence criterion is met, ultimately providing the maximum likelihood estimate of the parameters. The iteration terminates when the difference between the parameter vectors $\Theta^{k}$ and $\Theta^{k+1}$ is less than a predefined threshold.

### 3.3. Dynamic Reliability Prediction Method

By combining the concepts of “Soft Failure” and “First Hitting Time”, for the Wiener process with random effects, the probability density function (PDF) and reliability function (RF) have been derived from Ref. [35]:

(50) $$f_{T}(t)=\frac{w}{\sqrt{2\pi t^{3}({\sigma_{\eta }}^{2}t+\sigma^{2})}}\exp\left\{-\frac{{\left(w-\mu_{\eta }t\right)}^{2}}{2t({\sigma_{\eta }}^{2}t+\sigma^{2})}\right\}$$

(51) $$R_{T}(t)=\Phi \left(\frac{w-\mu_{\eta }t}{\sqrt{{\sigma_{\eta }}^{2}t^{2}+\sigma^{2}t}}\right)-\exp(\frac{2\mu_{\eta }w}{\sigma^{2}}+\frac{2{\sigma_{\eta }}^{2}w^{2}}{\sigma^{4}})\times \Phi \left(-\frac{2{\sigma_{\eta }}^{2}wt+\sigma^{2}(\mu_{\eta }t+w)}{\sigma^{2}\sqrt{{\sigma_{\eta }}^{2}t^{2}+\sigma^{2}t}}\right)$$

where w denotes a given safety threshold value; Φ(·) denotes the standard normal cumulative distribution function.

The expectation of remaining useful life (RUL) can be expressed, as follows:

(52) $$E(T)=\frac{\sqrt{2}w}{\sigma_{\eta }}D\left(\frac{\mu_{\eta }}{\sqrt{2}\sigma_{\eta }}\right)$$

where $D(z)=\exp(-z^{2})\int_{0}^{z}\exp(x^{2})dx$ represents the Dawson integral concerning the real number z.

$t_{i}$ represents the i-th time of sampling when the device is functioning properly. The real-time reliability at moment t is expressed using conditional reliability [36]:

(53) $$R(t|t_{i})=\frac{R(t)}{R(t_{i})}.$$

Let $x_{i}$ represent the observed value of performance degradation at moment $t_{i}$, and let w denote the set predefined threshold. There is

(54) $$\begin{array}{ll} R(t|t_{i}) & =P(x(t)<w|x_{i})=P(x(t)-x_{i}<w-x_{i})\\ & =P(x(t-t_{i})<w-x_{i}) \end{array}.$$

From (50) and (51), the device’s real-time PDF and RF can be obtained, as follows:

(55) $$f_{T|x_{i}}(t|x_{i})=\frac{w-x_{i}}{\sqrt{2\pi {\left(t-t_{i}\right)}^{3}({\sigma_{\eta }}^{2}(t-t_{i})+\sigma^{2})}}\exp\left\{-\frac{{\left(w-x_{i}-\mu_{\eta }(t-t_{i})\right)}^{2}}{2(t-t_{i})({\sigma_{\eta }}^{2}(t-t_{i})+\sigma^{2})}\right\}$$

(56) $$\begin{array}{l} R_{T|x_{i}}(t|x_{i})=\Phi \left(\frac{w-x_{i}-\mu_{\eta }(t-t_{i})}{\sqrt{{\sigma_{\eta }}^{2}{\left(t-t_{i}\right)}^{2}+\sigma^{2}(t-t_{i})}}\right)-\exp(\frac{2\mu_{\eta }(w-x_{i})}{\sigma^{2}}+\frac{2{\sigma_{\eta }}^{2}{\left(w-x_{i}\right)}^{2}}{\sigma^{4}})\\ \;\;\;\times \Phi \left(-\frac{2{\sigma_{\eta }}^{2}(w-x_{i})(t-t_{i})+\sigma^{2}(\mu_{\eta }(t-t_{i})+w-x_{i})}{\sigma^{2}\sqrt{{\sigma_{\eta }}^{2}{\left(t-t_{i}\right)}^{2}+\sigma^{2}(t-t_{i})}}\right). \end{array}$$

Form (52), RUL is given, as follows:

(57) $$E(T)=\frac{\sqrt{2}(w-x_{i})}{\sigma_{\eta }}D\left(\frac{\mu_{\eta }}{\sqrt{2}\sigma_{\eta }}\right).$$

Based on the online monitoring data of the equipment, the real-time stiffness degradation coefficients of the system are initially estimated using UKF. Subsequently, the parameters of the degradation model are estimated through the EM algorithm. Finally, based on the predefined performance degradation threshold, the probability density function and reliability function of the real-time remaining life are derived, enabling the dynamic real-time assessment of structural reliability.

## 4. Simulation and Discussion

### 4.1. Simulation Description

**Figure 1:**
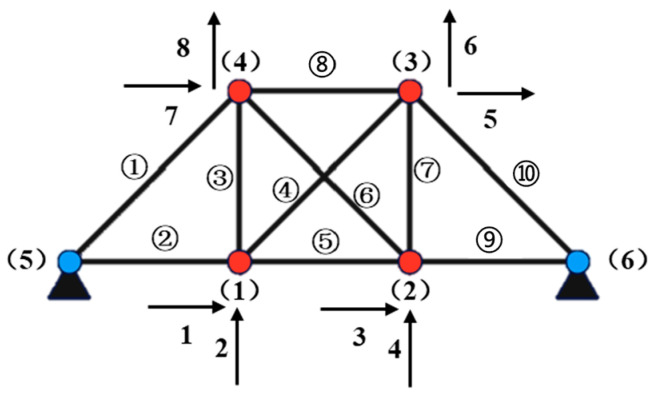
Planar truss structure schematic diagram. Red circles represent movable hinges, and blue circles represent fixed hinges. The numbers in brackets represent the node numbers, the numbers inside the circles represent the rod element numbers, and the remaining numbers represent the degree of freedom numbers.

A planar truss structure is shown in Figure 1. The finite element method is employed to establish the overall mass matrix and stiffness matrix of the structure. In this paper, using the diagonal mass matrix, the element mass matrix for the rod in the local coordinate system is

(58) $$M^{e}=\frac{m}{2}\left[\begin{array}{cccc} 1 & & & 0\\ & 1 & & \\ & & 1 & \\ 0 & & & 1 \end{array}\right].$$

The element stiffness matrix in the local coordinate system is

(59) $$K^{e}=\frac{EA}{l}\left[\begin{array}{cccc} 1 & 0 & -1 & 0\\ 0 & 0 & 0 & 0\\ -1 & 0 & 1 & 0\\ 0 & 0 & 0 & 0 \end{array}\right].$$

For the damping matrix, using the Rayleigh model, we have

(60) C=αM+βK

where α and β are constant coefficients.

### 4.2. Damage Parameter Estimation

**Figure 2:**
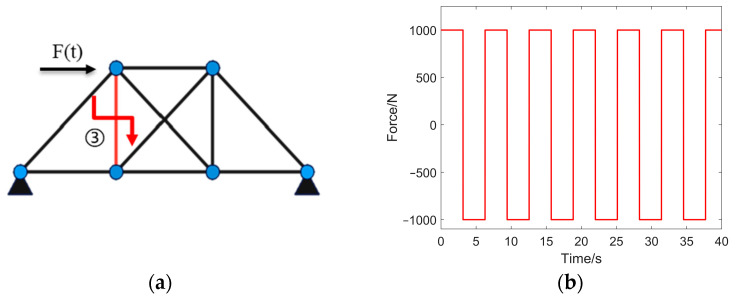
Structural damage simulation experiment. (**a**) Structural dynamics simulation diagram; (**b**) square wave force variation graph.

As shown in Figure 2a, damage has occurred in rod no. 3. The damage coefficient is 50%, the initial value is set at 90% of the truth value, and 5% Gaussian noise is added. The square wave force, as shown in Figure 2b, is applied to the seventh degree of freedom. Specific parameters are detailed in Table 1.

**Figure 3:**
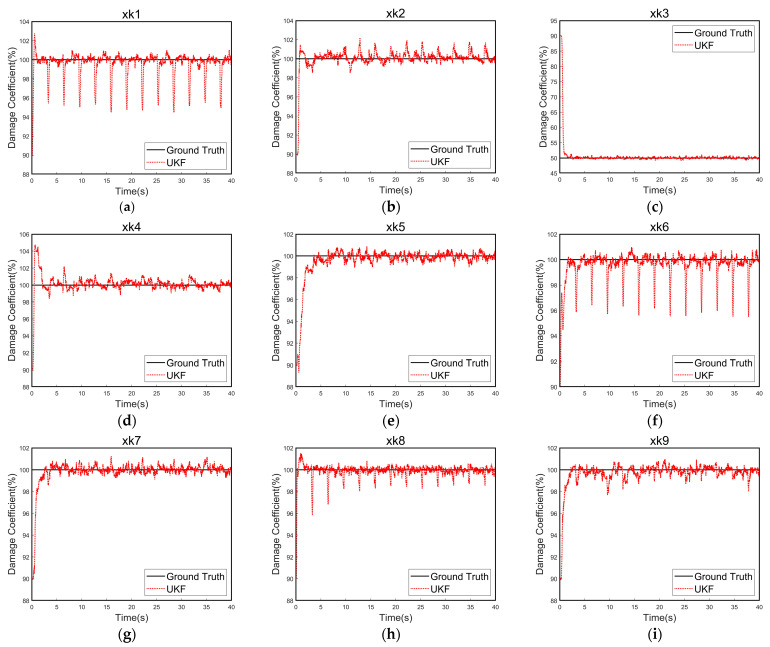
Stiffness damage coefficient identification curve. (**a**–**i**) The damage coefficient identification curves for rods 1 through 9, respectively.

Figure 3 illustrates that the damage identification method based on UKF can rapidly determine the stiffness coefficients of the structure. The results of the calculated stiffness damage coefficients for each rod are shown in Table 2. An analysis of the calculation results shows that the estimation error for each element stiffness damage parameter is minimal, confirming the validity of the method. We investigated the impact of noise on the accuracy of UKF-based damage identification. As noise levels increase, the estimation error of the damage coefficient grows, indicating that additional measurements may be needed to ensure accurate identification.

In addition, this paper compares the performance of KF, EKF, and UKF in structural damage identification. For nonlinear systems, KF fails to detect changes in the damage coefficient, while EKF’s identification accuracy is significantly lower than that of UKF, with a large relative error.

### 4.3. Model Parameter Estimation

**Figure 4:**
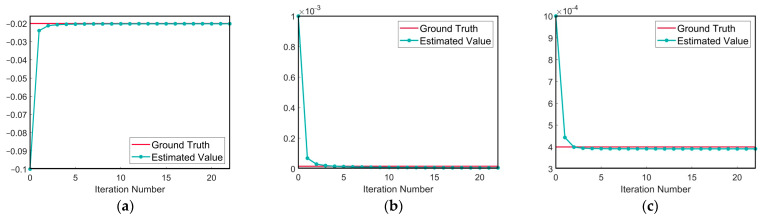
Parameter iteration convergence curves. (**a**) The iteration curve of $\mu_{\eta }$; (**b**) the iteration curve of ${\sigma_{\eta }}^{2}$; (**c**) the iteration curve of $\sigma^{2}$.

The EM algorithm is applied to estimate the model parameters for the stiffness damage coefficients identified in Section 4.2, and the relevant parameters are shown in Table 3. The iterative curve of the estimated parameters from the EM algorithm is shown in Figure 4, demonstrating that the algorithm converges rapidly.

**Figure 5:**
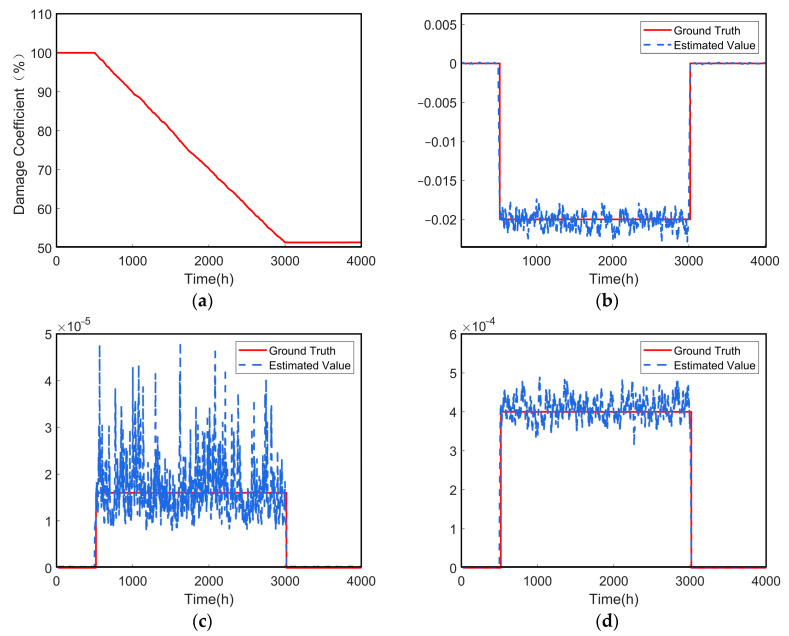
Identification of degradation process parameters. (**a**) The stiffness damage degradation process; (**b**) the estimation curve of $\mu_{\eta }$; (**c**) the estimation curve of ${\sigma_{\eta }}^{2}$; (**d**) the estimation curve of $\sigma^{2}$.

The stiffness damage degradation process is set up as shown in Figure 5a, with each degradation dataset containing 20 historical monitoring values. The dynamic estimation curves of $\Theta ={\left(\mu_{\eta },{\sigma_{\eta }}^{2},\sigma^{2}\right)}^{T}$ are shown in Figure 5b–d.

The mean and relative error of the parameter estimates are calculated as shown in Table 4. The errors may stem from factors such as the choice of initial values, local optima, data quality, and algorithm convergence issues. Additionally, since the parameter ${\sigma_{\eta }}^{2}$ is relatively small compared to the other two parameters, the estimated value by the EM algorithm fluctuates significantly, as shown in Figure 5b. To address this, techniques such as initial value optimization, global optimization algorithms, variational EM, incremental EM, and regularization methods can be employed to improve accuracy. The data in Table 4 indicate that the parameter estimates based on the EM algorithm closely align with the truth values, showing small relative errors. This demonstrates the feasibility and effectiveness of the method.

### 4.4. Real-Time Reliability Prediction

**Figure 6:**
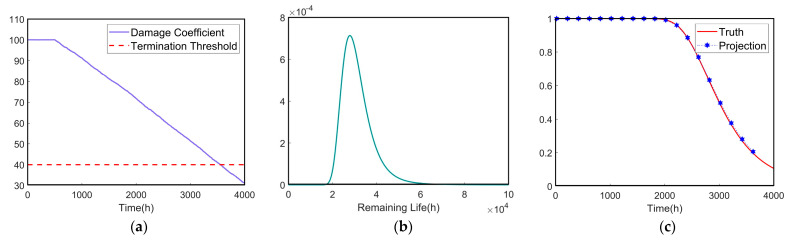
Reliability prediction simulation. (**a**) Description of stiffness damage degradation path. (**b**) Description of the PDF curve. (**c**) Description of the reliability prediction results.

Set the termination threshold w=40% for the stiffness degradation path shown in Figure 5a. From (32) and (33), we can calculate PDF and RF, as shown in Figure 6.

**Figure 7:**
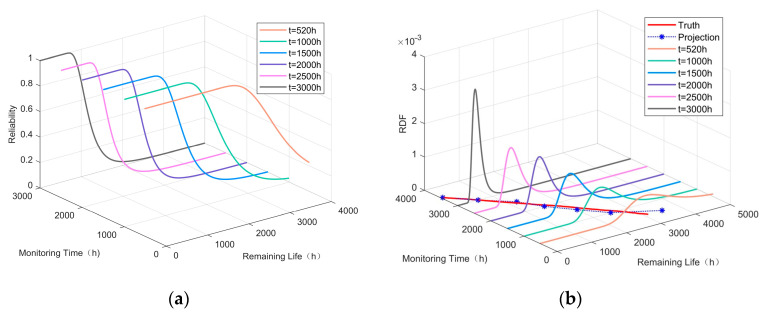
Real-time prediction. (**a**) Description of the dynamic reliability function curves. (**b**) Description of the remaining life prediction curves.

Using the dynamic reliability prediction method described in Section 3.3, we can calculate the dynamic reliability function curves and remaining life prediction curves for the damaged rod in the system at six time points: 520 h, 1000 h, 1500 h, 2000 h, 2500 h, and 3000 h. The results are shown in Figure 7.

As shown in Figure 7, the real-time reliability function of the stiffness decreases more significantly as the remaining life increases. By the time the equipment reaches 3000 h of use, its reliability has diminished to a very low level. From (39), we can calculate the estimated remaining life prediction for each monitoring time point, as shown in Table 5. The relative errors in the estimates of RUL at each monitoring time point are minimal compared to the truth values, which confirms the feasibility and validity of the entire method.

## 5. Conclusions

This paper, for the real-time reliability prediction of structures experiencing performance degradation, proposes a structural online damage identification and dynamic reliability prediction method based on the Unscented Kalman Filter (UKF). Specifically, the UKF estimates the dynamic state response and identifies damage parameters in a Wiener performance degradation model with random effects. The Expectation–Maximization (EM) algorithm is used to estimate model parameters with latent variables. By integrating the degradation model and conditional probability reliability, we derive the probability density and reliability functions to enable dynamic reliability prediction, supporting maintenance and decision-making. Simulation results validate the method’s feasibility and effectiveness, showing that it can quickly identify damage coefficients and provide accurate, real-time, dynamic reliability assessments.

Although this method has shown promising performance, it has several limitations, including reduced accuracy and computational dispersion in UKF estimation for strongly nonlinear problems, as well as significant computational errors and the risk of converging to local optima in the EM algorithm. Additionally, in real-time process monitoring, challenges such as untimely data collection and incomplete or distorted information must be addressed. Future research could explore the integration of intelligent optimization methods or data-driven models to enhance the algorithm’s robustness and optimization capabilities. Furthermore, multi-sensor data fusion and deep learning techniques could be employed to repair missing or distorted data, improving data integrity and accuracy, and better addressing uncertainties and challenges in complex environments.

## Figures and Tables

**Table 1:** Simulation parameters.

Parameter Name	Parameter Value
Structural model parameter	$l=10,A=0.16,E=2\times 10^{7},\rho =2700$
Rayleigh damping parameter	α=0.2,β=0.0001
Stiffness damage coefficient	$xk_{1}=xk_{2}=xk_{4}=xk_{5}=xk_{6}=xk_{7}=xk_{8}=xk_{9}=xk_{10}=100$
	$xk_{3}=50$

**Table 2:** Damage coefficient results for each element.

Element	1	2	3	4	5	6	7	8	9	10
Truth	100	100	50	100	100	100	100	100	100	100
Estimation	99.55	100.00	50.59	100.17	99.62	99.51	99.84	99.87	99.64	100.00
Relative Error (%)	0.45	0.004	1.19	0.17	0.38	0.49	0.16	0.13	0.36	0.001

**Table 3:** Model relevant parameters.

Parameter Name	Parameter Value
Truth Value	$\mu_{\eta }=-0.02,\sigma_{\eta }=0.004,\sigma =0.02$
Initial Value	$\mu_{\eta }=-0.1,\sigma_{\eta }=0.001,\sigma =0.001$
Observation Interval	Δt=1
Termination Threshold	$\vartheta =1\times 10^{-6}$

**Table 4:** Parameter estimation results of the degradation process.

Parameter	$\mu_{\eta }$	${\sigma_{\eta }}^{2}$	$\sigma^{2}$
Truth	−0.02	1.6 × 10^−5^	0.0004
Estimated Mean	−0.019904	1.6339 × 10^−5^	0.00040679
Relative Error (%)	0.48	2.12	1.698

**Table 5:** Remaining life prediction for each monitoring time point.

Monitoring Time (h)	520	1000	1500	2000	2500	3000	3500
Truth (h)	3118	2618	2119	1607	1045	549	72
Estimation (h)	3152	2611	2132	1555	1093	532	74
Relative Error (%)	1.09	0.27	0.61	3.24	4.59	3.10	2.78

## Data Availability

Data are contained within the article.
